# A Review of Boron Neutron Capture Therapy: Its History and Current Challenges

**DOI:** 10.14338/IJPT-22-00002.1

**Published:** 2022-06-09

**Authors:** Will H. Jin, Crystal Seldon, Michael Butkus, Wolfgang Sauerwein, Huan B. Giap

**Affiliations:** 1 Department of Radiation Oncology, Jackson Memorial Hospital/Sylvester Comprehensive Cancer Center, University of Miami Health Systems, Miami, FL, USA; 2 Deutsche Gesellschaft für Bor-Neutroneneinfangtherapie (DGBNCT), Universitätsklinikum Essen, Essen, Germany; 3 Department of Radiation Oncology, Nancy N. and J. C. Lewis Cancer & Research Pavilion, Savannah, GA, USA

**Keywords:** BNCT, neutron capture, particle therapy, neutron therapy, boron

## Abstract

**Mechanism of Action:**

External beam, whether with photons or particles, remains as the most common type of radiation therapy. The main drawback is that radiation deposits dose in healthy tissue before reaching its target. Boron neutron capture therapy (BNCT) is based on the nuclear capture and fission reactions that occur when ^10^B is irradiated with low-energy (0.0025 eV) thermal neutrons. The resulting ^10^B(n,α)^7^Li capture reaction produces high linear energy transfer (LET) α particles, helium nuclei (^4^He), and recoiling lithium-7 (^7^Li) atoms. The short range (5-9 μm) of the α particles limits the destructive effects within the boron-containing cells. In theory, BNCT can selectively destroy malignant cells while sparing adjacent normal tissue at the cellular levels by delivering a single fraction of radiation with high LET particles.

**History:**

BNCT has been around for many decades. Early studies were promising for patients with malignant brain tumors, recurrent tumors of the head and neck, and cutaneous melanomas; however, there were certain limitations to its widespread adoption and use.

**Current Limitations and Prospects:**

Recently, BNCT re-emerged owing to several developments: (1) small footprint accelerator-based neutron sources; (2) high specificity third-generation boron carriers based on monoclonal antibodies, nanoparticles, among others; and (3) treatment planning software and patient positioning devices that optimize treatment delivery and consistency.

## Mechanism of Action

**Figure 1.**
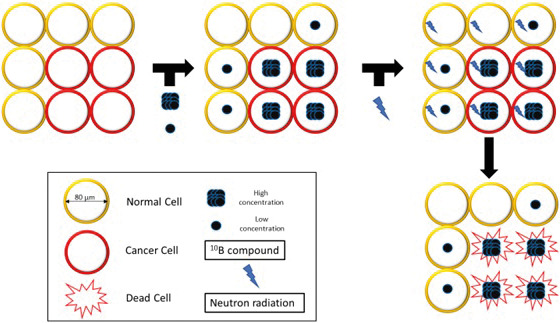
Extracellular mechanism of boron neutron capture therapy.

The underlying principle of all radiation therapy is to deliver a tumoricidal radiation dose to tumor while minimizing exposure of normal organs. There are 3 clinical modalities of radiation therapy: external beam radiation therapy (EBRT), brachytherapy, and systemic radionuclide therapy. Each has its own mechanism of action, advantages, and disadvantages. Most cancers are treated with EBRT, with various types of ionizing radiation (electron, photon, proton, helium, carbon) and radiobiological effectiveness (RBE). Both EBRT and brachytherapy target cancer at the macroscopic level, based on radiographic imaging such as computed tomography (CT) or magnetic resonance imaging, while systemic radionuclide therapies carry radioactive isotopes to the cell via a patient's blood supply. Boron neutron capture therapy (BNCT) provides an alternative and unique approach by delivering charged particles at the intracellular level, directly inside the tumor. It biologically and physically targets tumors via a binary system that consists of 2 separate components to achieve its therapeutic effect. Each component is stable and nontumoricidal by itself but becomes highly lethal to cancer cells when combined. Capture cross-sectional areas of nuclei and nuclear reactions are measured in barns to quantify the probability of interactions between small particles. Chargeless neutrons pass through normal tissue like a phantom, as their only means of significant interaction is through nuclear capture by stable nuclei like ^1^H, ^14^N, and ^10^B. Living tissues contain plenty of ^1^H (0.33 barns) and ^14^N (1.7 barns) and their capture cross section is small enough that reactions with these elements do not occur commonly enough to produce lethal radiation damage to normal tissues. In contrast, ^10^B (3990 barns) is exceedingly rare in living tissues but is exponentially more reactive with neutrons. When boron is infused into a patient, this inert payload is delivered to tumor cells and normal cells but is preferentially retained in tumor cells. Over time, the tumor amasses a much higher concentration of boron than the adjacent normal cell, yet neither sees any harm (**Figure 1**). It is only when a neutron beam is fired into a cell harboring a high concentration of boron that the reaction occurs and the boron-containing cell will be damaged.

BNCT is based on the nuclear capture and fission reactions that occur when nonradioactive ^10^B is irradiated with slow (thermal) neutrons of the appropriate energy to yield excited ^11^B, which undergoes an instantaneous nuclear reaction to produce a high-energy α particle and a high-energy ^7^Li nucleus. The nuclear reaction is as follows:



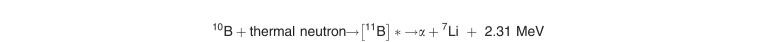




**Figure 2.**
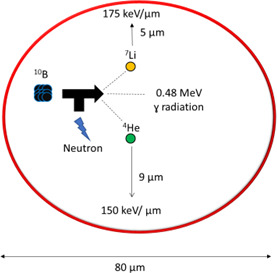
Intracellular mechanism of boron neutron capture therapy.

Alpha particles and lithium nuclei are charged particles with high linear energy transfer (LET) and RBE that produce closely spaced ionizations in the immediate vicinity of the reaction, with a range of 5 μm and 9 μm, respectively, while depositing dose with high LET at 175 keV/μm and 150 keV/μm, respectively (**Figure 2**). In reference, the diameter of an oral cavity squamous cell is approximately 80 μm. The range of tumoricidal radiation is limited within boron-containing cells. Therefore, successful BNCT is dependent on 2 factors: (1) the selective delivery of sufficient amounts of ^10^B to the tumor with much less to the normal tissues and (2) the availability of thermal neutrons at appropriate energy and quantity to trigger the reactions. Normal tissues can be spared from tumoricidal doses if they do not take up sufficient ^10^B or are not exposed to appropriate triggering neutrons.

## History

Transformative technologies are seldom produced by individuals. Rather, success is built on the stepwise iterations of their silent predecessors. BNCT is no exception. The earliest conceptual work for neutron capture therapy was first theorized in the mid-1930s by astrophysicist Gordon Locher. He hypothesized that neutrons emitted from a radium source captured by beryllium could be used to selectively kill tumor cells [1]. This was the first mention of “fast neutron capture.” Building upon this work, nuclear physicist Moritz Goldhaber discovered that the “slow neutron boron reaction” produced short and linear microscopic tracks in borax-filled photographs in 1935 [2]. Soon after, translational progress briefly stalled as global attention focused on World War II. The prioritization of armament bled into many scientific efforts. Arguably, BNCT was not one of them. Although costly, war accelerated critical advancements in nuclear physics that eventually brought a climactic end. The power of the atom was witnessed on the world stage, and a race to harness its capabilities began. Nuclear reactors built to study its destructive intent needed repurposing. Some reactors explored uses in alternative energy, while a few found their way to clinical medicine.

Neurosurgeon William Herbert Sweet and physicist Gordon Lee Brownell, of Massachusetts General Hospital and Massachusetts Institute of Technology (MIT), respectively, conducted the first clinical trials involving BNCT, using the Brookhaven Graphite Research Reactor in 1951 [3–5]. After performing the primary debulking craniotomy on patients with high-grade glioma in Cambridge, Massachusetts, Sweet transported them to Upton, New York, for the intravenous infusion of ^10^B-enriched borax (sodium tetraborate). Then, he exposed his patients to a single fraction of thermal neutrons from the experimental research reactor. None of the patients treated in these first clinical trials survived beyond 1 year and all experienced severe toxicities. Retrospective analysis suggested that subpar tumor to blood ratios (TBRs) with surgical debulking compromised the compartmentalization of boron, likely contributing to the poor outcomes seen in Sweet's initial series of patients with glioma [6]. It is important to note that thermal neutrons do not penetrate very deep; open craniotomies with exposed surgical cavities were required for adequate dose delivery.

Sweet's initial work and the awareness of his limitations led to collaborations to improve the selectivity of boronated compounds, as well as their delivery. Chemist Albert Herman Soloway joined the team and identified new boronated compounds with better pharmacokinetics. Simultaneously, pediatrician Lee Edward Farr joined the effort and brought his experience researching boronated compounds for clinical BNCT use at Brookhaven National Laboratory [4]. The trials continued. In 1960, Sweet infused intravenous paracarboxyphenylboronic acid into 16 patients with glioblastoma and treated them by using the MIT Research Reactor but was met with similarly unsuccessful results. Next, he delivered intra-arterial sodium decahydrodecaborate through the internal carotid artery in 2 patients. Autopsy revealed that these patients died from cerebral edema, surmised to have been caused by the combination of the surgical breakdown of the blood-brain barrier and intra-arterial infusion. Toxicities and unfruitful results coupled with the growing domestic fear in all projects “nuclear” halted BNCT progress in 1961. Similarly, the use of first-generation boronated compounds like boric acid and its derivatives declined.

As domestic interest waned, international efforts waxed. In 1968, Sweet's mentee, Hiroshi Hatanaka, started a Japanese clinical program. Decades later, Hatanaka [7] presented some of the most stellar clinical results ever reported in high-grade gliomas. While most of the patients treated had astrocytomas with a few glioblastomas, they reported a median survival of 21.3 months. In comparison, even with current standard-of-care management in glioblastomas, using craniotomy followed by adjuvant Stupp protocol radiation and temozolomide, median survival is 14.6 months [8]. These seemingly amazing results may have been driven by the enhanced pharmacokinetics of sodium borocaptate (BSH, Na_2_B_12_H_11_SH). EORTC 11961, a biodistribution study of BSH in patients with glioblastoma, revealed that the tumor to normal brain ratio was as high as 40 to 1 [9, 10].

Soon after, a second drug was introduced by Japanese dermatologist Yutaka Mishima. He experimented with perilesional injections of 4-borono-d,l-phenylalanine (BPA) in malignant melanomas, one of the first extracranial uses [11–16]. When other tumor histologic patterns were discovered to favorably take up BPA as well, it became the most widely used boronated compound. Then, fructose was conjugated to improve its hydrophilicity [16]. A shift in using the racemic form to the L-enantiomer occurred sometime in the early 1990s [12]. l-BPA, synthesized by John David Glass, would become the most prominent boronated compound in use for clinical trials [17]. Wittig et al [18] discovered active transport of BPA into cells through the l-amino acid transport system, which is highly regulated in most tumors, making BPA a drug that targets a wide variety of tumor entities.

Thus, a 30-year hiatus in BNCT use ended in the 1990s with the re-emergence of clinical programs at Brookhaven [19–22], MIT [23], Finland [24], European Union [25, 26], and Japan [27–28]. The arrival of the modern era in BNCT was marked by usage of second-generation compounds, l-BPA and BSH, but neutrons were still sourced from a reactor.

Prospective BNCT data remain sparse, with only 17 clinical trials ever registered with the National Institutes of Health. Head and neck cancers, melanomas, and glioblastomas were the only diseases studied prospectively. Of the 17 trials, 4 were terminated, 4 stopped updating, and 2 never published their results, leaving 7 complete studies [19, 28–32, 34]. Of note, EORTC 11961 was a phase I study that evaluated in vivo distribution of BSH [33], but phase II studies have yet to start. Extreme variability exists regarding protocols for the infused compound, the rate of infusion, the time between neutron irradiation, the anticipated blood boron concentrations, the time points at which the blood boron concentration is evaluated, and even the fractionation (**Table 1**).

**Table 1.** Prospective boron neutron capture therapy trials (nonmelanoma).

**Study**	**Pathology**	**Infusion timing**	**Mean TNTR fraction 1**	**Time on beam**	**Blood boron concentration**	**Blood monitoring intervals**	**Neutron source**
Kawabata 2009 phase II [30]	GBM	12 h before RT: 100 mg/kg BSH over 1 h; 6 h before RT: 700 mg/kg BPA over 6 h	N/A	N/A	N/A	N/A	Kyoto University Reactor OR JRR-4
Chadha 1998 phase I/II [19]	GBM	45 min before RT: BPA-f (100-250 mg/kg) over 2 h	3.5	45-65 min	13 μg/g	During and after infusion	Brookhaven
Wang 2016 phase I/II [31]	LRH&N	l-BPA-f (400 mg/kg) @ 180 mg/kg/h for 2 h, then @90 mg/kg/h during RT until beam off	3.4	23.1 min	28.8 ppm	1 h before, immediately before, immediately after infusion; 30 min after RT, before leaving reactor	Tsing Hua Open Pool Reactor
Kankaanranta 2012 phase I/II [29]	LRH&N	Before RT: l-BPA-f (400 mg/kg) + 10 mg cetirizine + 10 mg/d dexamethasone over 2 h	4.1	18.6 min	19.6 μg/g	Before, every 20 min during, after infusion; after first RT, after last RT	FiR1 Reactor
Kankaanranta 2007 phase I/II [34]	LRH&N	l-BPA-f (400 mg/kg) + 10 mg cetirizine + 10 mg/d dexamethasone over 2 h	2.5	60-120 min	20.9 μg/g	Before, every 20 min during, after infusion; after first treatment; after radiation	FiR1 Reactor
JHN002 phase II [28]	LRH&N	2 h before RT: borofalan (200 mg/kg/h for 2 h); during RT: borofalan (100 mg/kg/h)	3.5	43 min	32.8 ppm	Before infusion, then at 1 h, then at 2 h	CICS-1
JG002 phase II [32]	RMG	2 h before RT: borofalan (200 mg/kg/h for 2 h); during RT: borofalan (100 mg/kg/h)	3.5	NA^a^	25 ppm	Before infusion, then at 1 h, then at 2 h	Sumitomo BNCT30

**Abbreviations:** TNTR, tumor to normal tissue ratio; GBM, glioblastoma multiforme; RT, irradiation; BSH, sodium borocaptate; BPA, 4-borono-d,l-phenylalanine; N/A, not available; BPA-f, borophenylalanine fructose complex; LRH&N, locally recurrent head and neck cancers; ppm, parts per million; RMG, recurrent malignant glioma; Gy-Eq: Gray equivalent.

a Time on beam was not reported but was based on when the scalp dose reached 8.5 Gy-Eq.

The trials exploring BNCT in locally recurrent head and neck cancers (LRH&N) needed to navigate towards better toxicity profiles while maintaining adequate tumor control (**Table 2**). This cohort was first evaluated by the Helsinki group [29]. This group incorporated a novel 2-fraction BNCT with position emission tomography (PET)–based target delineation in a cohort that consisted primarily of squamous cell carcinomas, but also included 1 carcinosarcoma. Ultimately, 54% of patients experienced grade 3 or higher toxicities. Most commonly was mucositis (54%), followed by oral pain (54%), fatigue (32%), osteoradionecrosis (20%), xerostomia (20%), and soft-tissue necrosis (grade 4, 7%). The Taiwanese group [31] also evaluated 2-fraction BNCT in LRH&N. Their cohort consisted of mainly squamous cell carcinomas (64%), with a single carcinosarcoma (6%) and other histologic types (29%; adenocarcinoma, sinonasal carcinoma, undifferentiated carcinoma). They reported a 56% incidence of grade 3 or higher toxicities, mostly mucositis. The JHN002 trial [28] was the first to explore an epithermal accelerator-based neutron source (ABNS) in a recurrent head and neck cohort, specifically 38% squamous cell carcinomas and 62% non–squamous cell carcinomas (adenoid cystic carcinoma, acinic cell carcinoma, mucoepidermoid carcinoma, salivary ductal carcinomas, and mucosal malignant melanomas were all included in the non–squamous cell cohort). An additional point of novelty was the use of a single fraction, compared to the prior head and neck cohorts whose treatments were divided into 2 fractions to reduce potential toxicities. Other than hyperamylasemia, grade 3 or higher toxicities occurred in 24% of patients, a marked decrease from prior trials. Most importantly, this trial validated the use of an ABNS for BNCT use, which could potentially democratize the technique's use.

**Table 2.** Prospective nonmelanoma boron neutron capture therapy trial outcomes.

**Study**	**No. of patients**	**Minimum neutron irradiation dose to GTV or D80**	**Median follow-up, mo**	**Median overall survival , mo**	**Median PFS, mo**	**Response rate (PR + CR), %**	**G3+ toxicities, %**
Kawabata 2009 [30]	21	30 Gy-Eq (n = 10) or 40 Gy-Eq (n = 11, also received up to 30 Gy of external beam photons)	N/A	15.6	N/A	N/A	0
Chadha 1998 [19]	10	19.8-32.3 Gy-Eq	3-20.3 (range)	13.5	6	N/A	0
Wang 2016 [31]	25	Fraction 1: 13.0 Gy-Eq	19.7	24	N/A	64	29
		Fraction 2: 9.5 Gy-Eq					
Kankaanranta 2012 [29]	30	Fraction 1: 23 Gy-Eq	31	13	7.5	76	16
		Fraction 2: 22 Gy-Eq					
Kankaanranta 2007 [34]	12	Fraction 1: 21 Gy-Eq	14	13.5	9.8	58	42
		Fraction 2: 20 Gy-Eq					
		4-wk interval					
JHN002 [28]	21	Fraction 1: 31.1 Gy-Eq	31.2	2-Y: 58%	11.5	72	24
JG002 [32]	27	56.6 Gy-Eq	19	18.9	0.9	18.5^a^	33.30

**Abbreviations:** GTV, gross tumor volume; PFS, progression free survival; PR, partial response; CR, complete response; Gy-Eq: Gray equivalent; N/A, not available.

a 18.5% PR + CR assessed at the first 4-wk interval.

In patients with glioblastoma multiforme (GBM), the major hurdle for BNCT was not safety but efficacy. Glioblastoma multiforme, primary or recurrent, portends a dismal prognosis, and no treatment to date is considered curative. The Brookhaven group reported no severe toxicities in patients with GBM but descriptively had 3 patients experience post-BNCT seizures despite administering prophylactic high-dose steroids [19]. Median survival of 13.5 months was in line with standard-of-care Stupp protocol management of GBM. Their study was the first to explore epithermal neutrons, which have a partial skin-sparing effect, thereby no longer requiring scalp reflection and skull removal. Subsequently, the Japanese group [30] also reported no severe toxicities in patients with GBM but added EBRT after BNCT. Survival outcomes were also similar to standard-of-care management in GBM. Importantly, they found that there was a much higher survival benefit for all prognostic classes of GBM. In 2021, they incorporated SPM-011 as a new boronated agent and used an ABNS different from that used in JHN002. SPM-011 is a boron compound conjugated with phenylalanine, an amino acid. It is important to note that not all patients had recurrent malignant gliomas, where most were WHO (World Health Organization) grade IV GBM (85%) and ∼15% were grade II-III malignant gliomas [32]. Median overall survival was 18.9 months, significantly better than standard of care and expected median survival of 3 to 7 months, but 81.5% of patients experienced grade 3 or higher toxicities. Other than the 66.7% incidence of grade 3 hyperamylasemia, lymphopenia (14.8%) and brain edema (11.1%) were also seen. Especially in recurrent GBM, treatment-related edema is an impactful quality of life issue.

Currently, there is only 1 active trial listed that uses BNCT. A phase I clinical trial (NCT04293289) [35] is currently recruiting patients with angiosarcoma and malignant melanoma in Japan for the investigation of an investigational device, CIC-1, and an investigational drug, SPM-011. CIC-1 is the same ABNS used in JHN002. SPM-011 is the same boronated compound used in JG002 [32] and JHN002.

## Current Limitations and Future Directions

### Boronated Compounds

The ideal boronated compound must have high tumor to normal tissue selectivity and low toxicity to normal tissues. Selectivity must be achieved spatially (more in tumor than normal cells) as well as temporally (longer retention in tumor cells, rapid washout in normal cells). The goal is to get a TNTR greater than 4:1. Clinically, TBR is a quantifiable proxy for TNTR. Improvements to TBR can be achieved by discovering tighter affinity molecular conjugations, optimizing infusion timing in relation to neutron irradiation, and improving compartmentalization of boron. Currently, BSH and boronophenylalanine (BPA, l-4-dihydroxy-borylphenylalanine) are the only 2 agents used in clinical trials. There are currently 3 generations of boronated compounds. First-generation compounds included boric acid and its derivatives. These agents were only used by Sweet in the 1950s and 1960s. With meager tumor retention qualities and low TNTRs, the first-generation compounds were eagerly dropped for the next generation by the Japanese.

### Second Generation

Second-generation agents were more selective, TNTRs were >1, had less toxicity, and became the agents of choice after the 1960s. BPA recently became recognized as a “drug” by the Pharmaceuticals and Medical Devices Agency (PMDA, the Japanese equivalent of the US Food and Drug Administration) and is commercialized in Japan by Stella Pharma under the name STEBORONINE. With BSH, there were issues with heterogeneity in intercellular and intracellular concentrations within tumors and normal tissue, as well as low TBR. In patients with glioblastoma, experimental data showed that the TNTR was 3.8 when given at a dose of 25 mg/kg body weight and evaluated 3 to 7 hours after a 1-hour infusion [36]. However, the TBR was 0.27 in the same interval. The assumption that TNTR = TBR extracranially fell apart owing to the blood-brain barrier. BPA's TBR was better than that of BSH, but the TNTR was not equivalent to TBR. Goodman et al [36] and Koivunoro et al [37] reported that the ideal TNTR was 2, while the TBR was 3 at 200 minutes after infusion. Therefore, at least in GBMs, the blood-brain barrier remains a literal obstacle that must be overcome.

### Third Generation

Third-generation compounds are still experimental, but many seem promising. Biopharmaceutical screening studies yielded a plethora of nucleotide analogs, amino acids, liposomal conjugates, porphyrin derivatives, and conjugated targeted molecules [38, 39]. Borofalan, or SPM-011, is a conjugated amino acid that is in use in current trials, such as JHN002 and JG002. These compounds tend to favor molecular selectivity towards the targeted tumor cell, that is, the nucleus or DNA is targeted. The rationale is that lower concentrations of boron are required to produce a lethal effect if it is localized to the nucleus. However, without a compelling funding source or public health directive, the transition from preclinical studies to phase I/II clinical trials will be an arduous task.

### Theranostics for BNCT

Simultaneous tumor targeting and in vivo boron distribution in the body represents another development to produce optimized boron carriers for BNCT [40]. The multi-disciplinary nature of BNCT requires a diverse team proficient in pharmacology, pharmacokinetics, clinical radiation, dosimetry, medical physics, nuclear physics, and potentially surgery. BNCT can be regarded as both a biologically and a physically targeted type of radiation therapy.

With the latest wave of accelerators designed specifically for BNCT in a clinical setting, more centers are starting to implement patient positional systems, computerized dose calculation, image-based patient-specific treatment planning, and neutron beam spectrum. Like the infusion protocols seen in clinical trials, dosimetric or treatment planning approaches were institution dependent owing to institution-dependent neutron sources, types of boron carriers, and disease sites. An important difference to photon treatment planning is that in addition to slow neutrons, epithermal neutron beams also contain photons and fast neutrons. These mixed beam properties are calculated on the basis of previous nonhuman experimental RBE to determine their appropriate weighting [41]. The concept of the absorbed dose, used in conventional radiation therapy, is based on a homogeneous distribution of the deposited energy in the observed volume and cannot be applied to BNCT owing to the extremely high inhomogeneity in irradiating only tumor cells.

There are concerted efforts to advance BNCT from centers around the world through vendors and international societies such as International Atomic Energy Agency (IAEA), International Society of Neutron Capture Therapy, and Particle Therapy Cooperative Group BNCT Working Group. There is an emerging consensus to the treatment planning process as follows. After a patient is determined to be a candidate for BNCT, the patient is set up in treatment position (couch or chair) with an appropriate immobilization device (eg, head and neck thermoplastic mask for central nervous system or head and neck sites). Then the patient undergoes a blood-based biodistribution study using a boronated compound with metabolic imaging (such as 18F-FDG [18F-fluorodeoxyglucose]) with PET/CT scan. The biodistribution in tumor and normal structures is quantified with information from 3-D PET/CT imaging with the patient blood measurement. This information is then input into the computerized treatment planning software (TPS) to determine treatment parameters: injected dose, timing of the treatment, and specific neutron spectrum and flux. Current TPS uses Monte Carlo modeling to calculate dose distributions, dose-volume histograms, biological effects, and cumulative dose in the context of previous radiation treatments. It is assumed that blood concentration is calibrated to the PET/CT information, and the boron concentration in the tumor is predicted as based on the biodistribution study. On the day of treatment, the boronated compound is infused, then followed by neutron exposure with specific beam parameters (spectrum, flux, direction, duration). During the treatment, in vivo dose distribution can be monitored with a gamma camera or prompt photon detector. A reproducible model for prescribing and reporting this innovative radiation therapy modality will be needed [42].

### In Vivo Boron Concentration

An important factor that is often marked as a drawback in BNCT is the heterogeneous boron distribution within the tumor, which causes ambiguity in the calculated dose distributions. PET is capable of quantifying boron uptake [27, 31, 43]. In recent years, the pharmacologic and chemical behavior of the boron carrier using PET has become an additional stimulus for BNCT. 18F-BPA-PET scanned images were routinely used to clinically assess the distribution of boron molecules in patients by many investigators. In fact, PET with 18F-labelled BPA is used for patient selection in Japan [30]. In Finland, the use of 18F-BPA and PET was part of the inclusion criteria for BNCT patients [34].

### Potential Patients Treated

Which types of cancer are ideal BNCT candidates? Local regionally invasive malignancies, such as most solid malignant tumors, are good targets to start with. A special indication should be made for recurrent tumors that have already received full doses to organs at risk from conventional radiation therapy. Based on the results of the Helsinki group [29], recurrent head and neck cancers are ideal candidates. Given the lack of significant improvement in the poor prognosis of this histologic cancer type despite multi-modality treatments, glioblastomas are also an excellent choice. Finally, rare and/or highly radioresistant tumors, such as angiosarcomas, malignant melanomas, and meningiomas, may find roles for BNCT trials in the future.

BNCT is performed in 1, or at most, 2 fractions. This is an important issue for elderly patients or those with low performance scores, who may be intolerant of conventional 6-week fractionation schemes. Additionally, this extreme hypofractionation is critical for patients who travel far distances for medical tourism. While the treatment can be performed at the BNCT center, recruitment and follow-up can be done at a participating local cancer center.

### Neutron Source

In BNCT, neutrons must be delivered with high flow rate (kerma), the right energy, and minimal contaminants. Neutrons for epithermal radiation are generated from nuclear reactors and ABNSs. Most of the nuclear reactors commissioned in the 1950s are shut down today, on the verge of closure, or minimally active in BNCT trials; only a few remain open.

The single, largest barrier to BNCT adoption was having direct access to a nuclear reactor in proximity and this remains a huge disparity for equitable care. Conceptually, BNCT seems like the ultimate precision medicine solution. Yet, even the financial hurdles of starting a new clinical program were eclipsed by the logistical necessity of a nuclear reactor.

A change of paradigm occurred with the appearance of ABNS, which began seeing clinical use and validation at Kyoto University Research Reactor Institute by Sumitomo Heavy Industries Ltd, using a cyclotron and a beryllium target. Based on this experience, a hospital-based system was installed at the Southern Tohoku General Hospital in Koriyama (Fukushima Prefecture) and the same system is now treating patients at the Kansai BNCT Medical Center at Osaka Medical College. Another system using a linear accelerator and a lithium target is from Cancer Intelligence Care Systems, Inc. They are currently performing clinical trials for validation at the National Cancer Center Hospital in Tokyo. In Europe, 1 facility from Neutron Therapeutics Inc is under commission at Helsinki University Hospital and recently, the Italian government decided that the National Hadron Therapy Center in Pavia will obtain a BNCT facility. These new hospital-based accelerator systems promise a new area in cancer treatment in several countries with worldwide outreach. ABNS ranging from low-energy electrostatic machines to higher-energy cyclotrons and much-higher-energy Linacs or synchrotrons have been used. The neutron beam produced by ABNS has a low-intensity flux compared to nuclear reactor sources, but the possibility exists for delivering neutron sources with the desired intensities via many accelerators. Moreover, ABNS are compact, less expensive than nuclear reactors, and operationally similar to the Linacs in most radiation therapy departments.

There are currently 4 vendors providing accelerator technologies for BNCT: 2 from Japan and 2 from the United States. We do not have details of the 2 Japanese technologies in open literature. Sumitomo Heavy Industries Ltd describes their NeuCure BNCT system [44] that uses a cyclotron as the accelerator with a linear beam line and a beryllium target. Patients are treated on either a couch or chair, while planning is performed via their proprietary NeuCure Dose Engine TPS from Raysearch's Raystation. Cancer Intelligence Care Systems (CICS) is the second Japanese company that manufactures ABNS for BNCT [45]. Their Website describes their system as “…an accelerator-based neutron capture therapy device developed by CICS. It produces neutrons by bombarding a lithium target with protons accelerated by a Radio Frequency Quadrupole (RFQ) linear accelerator. CICS-1 is notable for the low level of contamination of fast neutrons, which are detrimental to the human body. The neutrons produced have a low energy level of 800keV or less, facilitating the miniaturization of the moderator used to slow the neutrons down to around 10keV, a level suitable for BNCT.”

**Figure 3.**
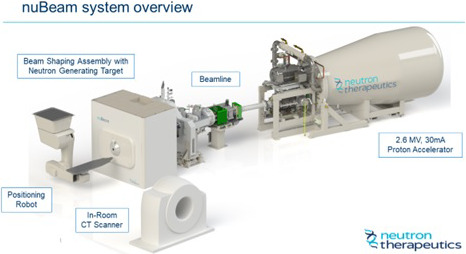
Artistic rendering of TAE Life Sciences' Alphabeam system. Abbreviation: CT, computed tomography.

In the United States, 2 innovative companies have identified the unmet need of a safe and reliable hospital-based neutron source for BNCT: Neutron Therapeutics and TAE Life Sciences. Neutron Therapeutics developed a room-sized ABNS platform, nuBeam, that uses a 2.6-MV, 30-mA proton accelerator to drive the neutron generation process (**Figure 3**) The proton beam is directed at a rotating solid-lithium neutron-generating target that distributes the 78-kW beam over a large area to ensure reliable operation. Helsinki University Hospital will be validating clinical use of this system in treating recurrent head and neck cancers. Installation of a second unit at Shonan Kamakura General Hospital in Kanagawa Prefecture, Japan, is underway.

**Figure 4.**
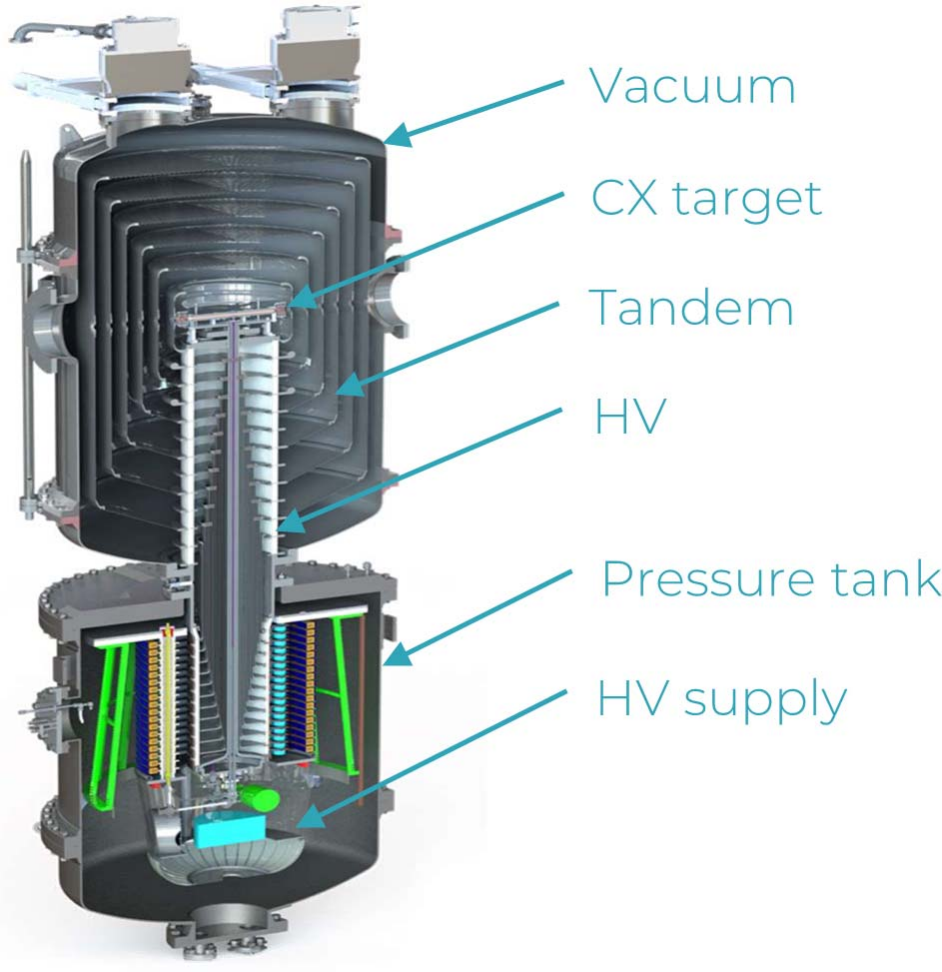
TAE Life Sciences Tandem Accelerator. Abbreviations: CX, Charge Exchange; HV, high voltage.

TAE Life Sciences offers both complete BNCT technologies and new boron drugs. Their Alphabeam system includes customizable neutron sources from a fixed beam with beam-shaping assembly, and ceiling-mount robotic couch for patient positioning. It is based on developments from the Russian Budker Institute [46, 47] into a state-of-the-art ABNS named Alphabeam. This system consists of a negative ion source, pre-accelerator, tandem accelerator, high-energy beam lines and neutron production targets as part of the neutron beam system. The tandem accelerator was developed specifically for BNCT and provides a compact and reliable device that delivers low-energy epithermal neutron beams optimized for clinical use in single or multiple room configurations. The neutron beam system ion source generates high currents (nominal 10 mA) of negative hydrogen ions (H−) that are accelerated to 150 keV in the pre-accelerator before entering a narrow port on the side of the tandem accelerator, which includes a series of nested metal shells and a high-voltage supply (**Figure 4**). Progressive voltage increases from the outer shell to roughly +1.2 MV at the innermost shell, accelerating the H− ions to about 1.35 MeV at the center, where a charge exchange component strips the ions of both electrons. This charge reversal allows the resulting protons to experience the same acceleration to the opposite side of the accelerator, resulting in a final energy of 2.5 MeV. High-energy beam line components direct the fully energized protons to the correct treatment room while maintaining beam focus. Lithium targets are bombarded by the proton beam, producing neutrons via the p + ^7^Li → n + ^7^Be reaction. TAE Life Sciences plans to install its first commercial ABNS in mainland China in 2021.

### Combined International Efforts

The results of the Japanese BNCT clinical trials, performed under the auspices of the PMDA, will likely spark a rapid growth in BNCT interest. Currently, the National Cancer Institute (NCI) created a working group for BNCT, in addition to PTCOG (Particle Therapy Co-Operative Group). The IAEA is also preparing a Technical Document (TECDOC) with the intention to replace the 20-year-old TECDOC related to BNCT at research reactors.

The German Society for BNCT started worldwide activities to prepare and coordinate the complex translational research activities necessary for the success of BNCT (RENOVATE). With its international partners, they developed a conceptual framework (“BNCT Global”) for how to rapidly introduce BNCT into mainstream oncology and make it a reimbursable modality [48]. There is a need for continued, strong, interdisciplinary clinical research in the form of an international reference center to support local BNCT hospitals focused on patient treatments. Ideally, this center will act as a partner for regulatory authorities worldwide, as well as a training center for clinicians, technicians, and scientists prepared to practice in their own local BNCT centers. Furthermore, this center will be in close exchange with preexisting BNCT facilities, which until now have not had strong scientific backup.

## Conclusion

Despite its decades long history, BNCT still has limitations in the way of widespread adoption. The availability of reliable neutron sources, boron conjugate development, and the cooperation of key stakeholders are currently being tackled by leaders in their respective fields. An International Reference Center for BNCT will need to be established. This center would coordinate the different clinical and nonclinical disciplines needed to perform the translational research required to introduce BNCT as a substantial treatment modality in oncologic practice.
